# Cutaneous lupus erythematosus after treatment with paclitaxel and bevacizumab for metastatic breast cancer: a case report

**DOI:** 10.1186/1752-1947-5-243

**Published:** 2011-06-27

**Authors:** Pia Vihinen, Outi Paija, Atte Kivisaari, Leena Koulu, Heikki Aho

**Affiliations:** 1 Department of Oncology and Radiotherapy, Turku University Hospital, PO Box 52, Fin-20521 Turku, Finland; 2 Department of Dermatology, Turku University Hospital, Turku, Finland; 3 Department of Pathology, Turku University Hospital, Turku, Finland

## Abstract

**Introduction:**

The monoclonal anti-vascular endothelial growth factor antibody bevacizumab is increasingly used in the treatment of several malignant tumors. The usual side effects of this drug are hypertension and proteinuria. Paclitaxel is widely used in the treatment of breast cancer and head and neck carcinomas. Neither of these two drugs typically causes skin disorders. Paclitaxel-related cutaneous lupus erythematosus has been described before, but in earlier cases patients had a history of autoimmune disease.

**Case presentation:**

We report a case of a 65-year-old Caucasian woman who presented with cutaneous lupus erythematosus after receiving paclitaxel-bevacizumab combination treatment as first-line therapy for metastatic breast cancer. Her cutaneous symptoms and increased serum anti-SSA and anti-SSB antibodies disappeared shortly after the discontinuation of therapy.

**Conclusion:**

We conclude that cutaneous lupus erythematosus can also be seen in patients without earlier anamnesis of autoimmune disorders and that, furthermore, bevacizumab might cause atypical cutaneous side effects.

## Introduction

Systemic lupus erythematosus (SLE) is an autoimmune disease characterized by the presence of autoantibodies to the nuclear and cytoplasmic antigens in conjunction with several clinical manifestations [1]. Cutaneous lupus lesions typically occur in light-exposed areas and can be triggered by sunlight exposure [1]. Drug-induced lupus erythematosus (DILE) is a syndrome that shares symptoms and laboratory characteristics with idiopathic SLE [2]. More than 80 drugs have been associated with DILE [2]. Paclitaxel is an anti-cancer agent that is used for the treatment of patients with breast cancer, ovarian cancer, gastrointestinal cancers and tumors of the head and neck. Paclitaxel treatment is often associated with neurological pain, hair loss and nail changes, but skin disorders such as photosensitivity are less common. Paclitaxel has been associated with inducing acral erythema [3], scleroderma [4] and Stevens-Johnson syndrome [5]. A recent case report also described paclitaxel-induced cutaneous lupus erythematosus in patients with Sjögren's syndrome [6].

Bevacizumab is an anti-vascular endothelial growth factor (anti-VEGF) antibody that may improve the effect of taxane-based regimens in the treatment of metastatic breast cancer [7]. A recent study has shown that bevacizumab-paclitaxel combination therapy prolongs progression-free survival, compared with paclitaxel alone, in patients with metastatic breast cancer [8]. The most common toxicities associated with bevacizumab are hypertension and hemorrhage, gastrointestinal perforation, arterial thromboembolism, impaired wound healing and proteinuria [9]. Cutaneous disorders are rare side effects of bevacizumab therapy. Cutaneous side effects were not mentioned at all in an earlier study in which 365 patients were treated with bevacizumab-paclitaxel combination therapy, and the overall frequency of grade 3 allergic reactions in that study was only 3% [8]. In the present case report, we describe a patient without known previous autoimmune disorders who developed a reaction resembling acute cutaneous lupus erythematosus (LE) after therapy with paclitaxel and bevacizumab.

## Case presentation

Our patient was a 58-year-old Caucasian woman who had been diagnosed in September 1999 with estrogen receptor-positive (ER^+^), progesterone receptor-positive (PR^+^), human epidermal growth factor receptor 2/neu (Her2/neu)-negative ductal breast cancer assessed as American Joint Committee on Cancer stage IIA (pT1 pN1 M0 G1). She was initially treated with partial mastectomy and evacuation of axilla. No signs of disseminated disease were detected. Radiotherapy (50 Gy) was given to the left breast and lymph nodes. The patient received adjuvant tamoxifen therapy (20 mg/day) for five years, until January 2005. In 2003, she was diagnosed with hypothyroidism and treated with thyroxin substitution daily. In 2004, she was diagnosed with high blood pressure and was treated with metoprolol (47.5 mg/day).

In March 2007, routine mammography showed a new local tumor in the left breast, and radical mastectomy was performed. The ductal residual tumor was assessed as pT1 pNX G2 and was ER^+^, PR^+ ^and Her2/neu-negative. A palpable tumor was found at the left side of her neck, and a fine-needle biopsy showed metastasis of her breast cancer. A whole-body computed tomographic scan showed multiple liver metastases and multiple metastases in the left lung and the spleen.

**Figure 1:**
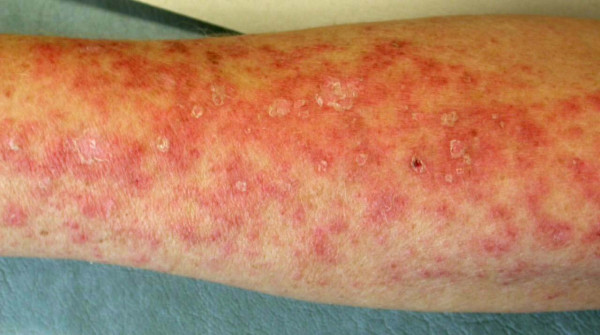
**Patient's forearm on day 36 after beginning paclitaxel-bevacizumab combination therapy**.

**Figure 2:**
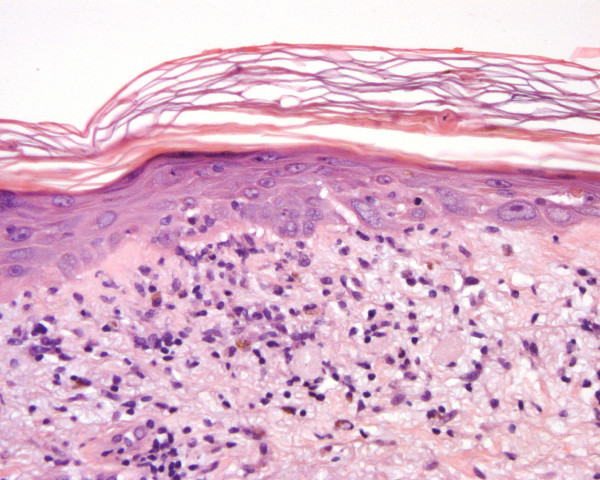
**Inter-phase dermatitis in skin biopsy taken from the patient's forearm**. Basal degeneration as well as some regeneration is seen in epidermal keratinocytes. An apoptotic dyskeratotic cell is present in the horn layer. A slight lymphocytic reaction and some melanophages were found in the papillary dermis. An occasional lymphocyte was seen in the epidermis (hematoxylin and eosin stain; original magnification, × 250).

First-line chemotherapy was started with weekly paclitaxel 80 mg/m^2 ^on days 1, 8 and 15 of a 28-day cycle and concomitant bevacizumab 10 mg/kg every two weeks. Her blood pressure was elevated after the first infusion, and the previous metoprolol dose was doubled to 90 mg/day. Her serum creatinine and bilirubin levels were normal (creatinine 77 μmol/L, normal range 50 to 90 μmol/L; bilirubin 18 μmol/L, normal range 5 to 25 μmol/L) before beginning therapy. Her serum alkaline phosphatase level was increased (214 U/L, normal range 35 to 105 U/L). After two combined infusions of paclitaxel-bevacizumab, an itchy papulosquamous rash was apparent on sun-exposed areas of the skin of her arms, legs and face (Figure 1). The rash was treated with cetirizine (10 mg/day) and topical corticosteroids. Her blood pressure was further elevated, and metoprolol was replaced by candesartan cilexetil-hydrochlorothiazide combination therapy. Her paclitaxel-bevacizumab treatment continued, but the rash on her arms and legs worsened. The patient was referred to a dermatologist, and skin biopsies were performed. The skin biopsy specimen showed non-specific inter-phase dermatitis, which may be associated with LE (see Figure 2). A direct immunofluorescence study did not show deposition of immunoglobulins at the basement membrane zone, but C3 on Civatte bodies was positive. Simultaneously, her serum anti-SSA/Ro (> 240 U/mL, normal range 0 to 6.99 U/mL), anti-SSB/Ro (94.4 U/mL, normal range 0 to 6.99 U/mL) and anti-extractable nuclear antigen (anti-ENA) antibodies were positive. Paclitaxel-bevacizumab combination therapy was discontinued and replaced by cyclophosphamide, epirubicin, fluorouracil (CEF), after which her skin rash disappeared within two weeks. Her serum anti-SSA/Ro antibodies were 8.1 U/mL and her anti-SSB/Ro antibodies were 5.0 U/mL when checked three months after discontinuation of the therapy. Her serum anti-ENA antibodies were not checked. Her serum alkaline phosphatase level had decreased during therapy (from 274 U/L at maximum to 121 U/L, normal range 35 to 105 U/L), and her other liver enzyme values were not markedly changed.

## Discussion

Common presenting symptoms of DILE include arthralgia, myalgia, malaise and fever [2]. The laboratory profile of DILE includes anti-histone antibodies, especially immunoglobulin G anti-DNA antibody, in the absence of other anti-nuclear antibody specificities [2]. Typically, the symptoms improve within days or weeks after discontinuation of the suspected drug. DILE with cutaneous manifestations has rarely been described in patients treated with paclitaxel [6] or docetaxel [10]. In earlier studies, taxane-related cutaneous LE has been related to a pre-existing history of Sjögren's syndrome [6]. Our patient had no history of autoimmune disorders; however, hypothyroidism was present. This might be associated with the previous adjuvant radiotherapy for local breast cancer.

Skin acneiform rash is a typical side effect of cetuximab but has seldom been described in association with bevacizumab. However, Gotlib and co-workers [11] described a patient with colorectal cancer who developed a skin rash secondary to bevacizumab therapy that correlated with response. In our patient, cutaneous toxicity was apparent only in sun-exposed areas, that is, the face, forearms and legs, in contrast to the more commonly seen acneiform rash on the face, neck and upper back of patients treated with cetuximab [12].

In our case, the cutaneous biopsy showed inter-phase dermatitis (Figure 2), which is a non-specific cutaneous reaction to several stimuli. It is seen in lichen planus, other lichenoid reactions, erythema multiforme and LE, all of which can be caused by drugs. Typical lichen planus can be ruled out morphologically and clinically. The histological pattern matches that of erythema multiforme or subacute LE. Positive anti-SSA/Ro antibodies support the latter possibility. However, direct immunofluorescence with positive C3 in the dermoepithelial junction was non-specific and the typical lupus band was not observed.

Cutaneous LE has not previously been described in patients treated with bevacizumab. However, previous studies involving skin specimens have shown that receptors for VEGF are present in keratinocytes in human epidermis [13]. Similarly, Kikuchi and co-workers [14] have shown that serum concentrations of VEGF are increased in collagenous diseases, suggesting that this growth factor might also be important in the pathogenesis of collagenous diseases other than rheumatoid arthritis. In our case, it is probable that the cutaneous reaction resembling LE was caused by paclitaxel, but the role of bevacizumab cannot be ruled out. We suggest that this skin reaction was specifically related to these drugs because it did not reappear when the patient received CEF treatment, even though fluorouracil is known to be a photosensitizing agent which can also induce DILE [2].

## Conclusions

We conclude that paclitaxel-bevacizumab combination treatment might cause cutaneous LE in patients with no history of autoimmune disorders. In addition, while the use of bevacizumab for various indications such as metastatic renal cancer, colorectal cancer, lung cancer and breast cancer is increasing, rare side effects such as skin disorders are possible.

## Abbreviations

CEF: cyclophosphamide, epirubicin, fluorouracil; CT: computed tomography; DILE: drug-induced lupus erythematosus; LE: lupus erythematosus; VEGF: vascular endothelial growth factor.

## Consent

Written informed consent was obtained from the patient for publication of this case report and any accompanying images. A copy of the written consent is available for review by the Editor-in-Chief of this journal.

## Competing interests

The authors declare that they have no competing interests.

## Authors' contributions

PV was the major contributor to the writing of the manuscript. PV and OP participated in the treatment of the patient as medical oncologists and analyzed and interpreted the patient data regarding treatment of metastatic breast cancer. AK and LK participated in the treatment of the patient's skin symptoms and performed the skin biopsy. HA performed the histological examination of the skin biopsy. All authors read and approved the final manuscript.
